# Effects of non-medical health coaching on multimorbid patients in primary care: a difference-in-differences analysis

**DOI:** 10.1186/s12913-019-4367-8

**Published:** 2019-08-22

**Authors:** Vishalie Shah, Jonathan Stokes, Matt Sutton

**Affiliations:** University of Manchester, Manchester, UK

**Keywords:** Health coaching, Task-shifting, Multimorbidity, Population health, Chronic disease, Primary care, Workforce, Prevention strategies, Self-management, Difference-in-differences

## Abstract

**Background:**

Health systems, globally, are attempting to strengthen primary care to promote a population-health management approach to care provision, incentivising prevention and self-management. This paper evaluates the "Enhanced Primary Care" model implemented in a geographical region in England. Enhanced Primary Care introduces a new non-medical role, health coaches, to the traditional primary care team to provide additional support for patients with chronic conditions. We evaluate effects of health coaching on patient outcomes using a quasi-experiment.

**Methods:**

We estimate the programme’s effects on health status (EQ-5D-5L, physical functioning, psychological wellbeing, and resilience), health behaviour (smoking habit), experience of care (person-centeredness and continuity of care), and health care (primary care) utilisation using data from 3.5 million respondents to the national GP Patient Surveys between 2013 and 2017.

We use a weighted difference-in-differences design to compare changes in outcomes over time between intervention practices and comparable control practices in the rest of England. We conduct our main analysis on multimorbid patients and additional analysis on all patients to assess population-level effects.

**Results:**

For multimorbid patients, we find reductions in psychological wellbeing (short and medium term) of −0.0174 (95% confidence interval −0.0283 to −0.0065), relative difference −2% from the pre-intervention mean; and person-centeredness (short term) of −0.0356 (−0.0530 to −0.0183), −4%. We find no significant effects on other outcome measures. For population-level effects, in the short term we find reductions in primary care utilisation of −0.0331 (−0.0448 to −0.0214), −5%. All other outcomes are not consistently statistically significant.

**Conclusions:**

Our results show that there is very little effect of health coaching on patient experience and outcomes in the short-to-medium term (up to 14 months). Introduction of Enhanced Primary Care was associated with slightly lower psychological wellbeing and person-centeredness amongst multimorbid patients (it might be initially difficult for patients to adjust to the model). However, it was also associated with a decline in primary care visits at the population-level (potentially freeing up practitioner time for more complex patients). The results raise important questions regarding primary care workforce changes advocated in the NHS Long Term Plan, and the time horizon of any benefits of prevention strategies.

**Electronic supplementary material:**

The online version of this article (10.1186/s12913-019-4367-8) contains supplementary material, which is available to authorized users.

## Background

Health systems, globally, are facing unprecedented pressure from changing demographics. Patients with chronic conditions account for approximately half of all general practice (GP) appointments in the UK [1]. Preventable ill-health is responsible for an estimated 40% of the burden on all health care services in England [2]. The rising prevalence of multimorbidity – the co-existence of two or more chronic conditions [3] - has shifted the policy rhetoric globally towards prevention and self-management support [4–7] Given the benefits of a robust GP gatekeeper, new models of care and the primary care skill-mix required to deliver these have become the most recent policy focus [8, 9], including in the global Astana Declaration and the NHS Long Term Plan [10, 11]. Furthermore, the shortage of primary care doctors to cope with this growing demand has become a key challenge for policymakers [12]. This has led to strategies designed to increase the primary care workforce through “task shifting”, by adding new, non-medical roles to the traditional primary care team [13, 14].

In this paper, we investigate a new model of care implemented in a geographical region in England, The South Somerset Symphony Programme. The region has redesigned primary care provision to focus on prevention and management techniques for chronic disease patients, as well as specialist care for complex cases [15]. We evaluate the “Enhanced Primary Care” component of The Symphony Programme. Enhanced Primary Care provides additional prevention and self-management education support for patients with multimorbidity through the introduction of a new, non-medical role, “the health coach”. This model aims to up-skill the workforce by allowing GPs to focus on the most severe patients, whilst health coaches assume responsibility for chronic disease management. This is a similar role to the social prescribing link worker promoted for investment in the recent NHS Long Term Plan [16]. Health coaching aims to promote patient activation by shifting the balance of responsibility from care provider to patient [17–19], and empowering them to make important behavioural and lifestyle changes [20, 21]. Enhanced Primary Care is based on a similar model implemented in the US by Iora Health [22]. A key distinction between the US and UK models is that health coaches in the US tend to have previous clinical experience (e.g. nursing), whereas those in the UK typically have a background in administration or social work. This study fills a gap in the evidence by exploring health coaching by non-clinicians, which might provide different effects moderated by both practitioner ability and patient acceptability.

The evidence base for health coaching in the UK is limited. Apart from small-scale studies conducted in the East of England [23–26], the majority of rigorous evidence originates from abroad (including the US, Canada and Finland) [27–32]. Existing research tends to evaluate health coaching from clinicians’ perspectives, with mixed overall results on health outcomes and behaviours. There are few evaluation studies from patients’ perspectives, as well as the impact on primary care utilisation. We have previously gathered qualitative evidence on Enhanced Primary Care and found that while health coaching enabled more frequent, proactive contact between the GP practice and the patient, some patients did not adjust to this new model of care [15]. This work aims to build on our qualitative results with a rigorous quantitative evaluation of health coaching using quasi-experimental methods. Using national individual-level data from the General Practice Patient Survey (GPPS), we estimate the impact of Enhanced Primary Care on outcomes across the causal chain - experience of care, health behaviour, health status and health care utilisation - in the short-to-medium term (up to 14 months) [33].

### The South Somerset Symphony Programme – Enhanced Primary Care

The intervention was introduced in South Somerset, located in the South West of England. South Somerset has a population of approximately 165,000 and comprises 19 GP practices, 4 community hospitals, 1 community mental health team and 1 district general hospital [34, 35].

The Symphony Programme was launched in 2015, aimed at developing a model of integrated care spanning the entire geographical population, but focussed on patients with chronic conditions. The region received special project funding, as part of NHS England’s Five Year Forward View [8], to become a Primary and Acute Care Systems vanguard [36]. Through the introduction of two new interventions, the programme planned to increase synergies across the health care system. The first intervention is the Complex Care Hub, which supports high-risk patients through case management by multidisciplinary teams, achieved through increased co-ordination between health care providers. This paper looks to evaluate the second, more novel and prevention-oriented intervention: Enhanced Primary Care (see Table 1).

**Table 1:** What is Enhanced Primary Care?

What is Enhanced Primary Care?	A new model of primary care that aims to upskill the workforce by adding a new non-medical role to the primary care team: health coaches
Target group of Enhanced Primary Care	Patients with at least one chronic condition (~ 18% of the population in South Somerset)
Role of the health coach	To act as a single point of contact for patients; and to provide additional self-management and education support for patients
Background of the health coach	Typically from an administration/receptionist background, but some practices have also hired former social workers
Training of the health coach	All health coaches receive a two-day training course in health coaching. Health coaches are trained in different techniques to improve person-centeredness by supporting patients in identifying and achieving their goals
Number of GP practices that have implemented Enhanced Primary Care	17 out of 19 practices in South Somerset, with some at a more advanced stage than others. The scheme has been rolled out in three waves across the region

Source: Stokes J, Cheraghi-Sohi S, Kristensen S. R, Sutton M. Work Package 2: Thick descriptions of – South Somerset Symphony Programme. 2016; Available at: https://www.selfie2020.eu/wp-content/uploads/2016/12/SELFIE_WP2_UK_Final-thick-descriptions.pdf

**Fig. 1:**
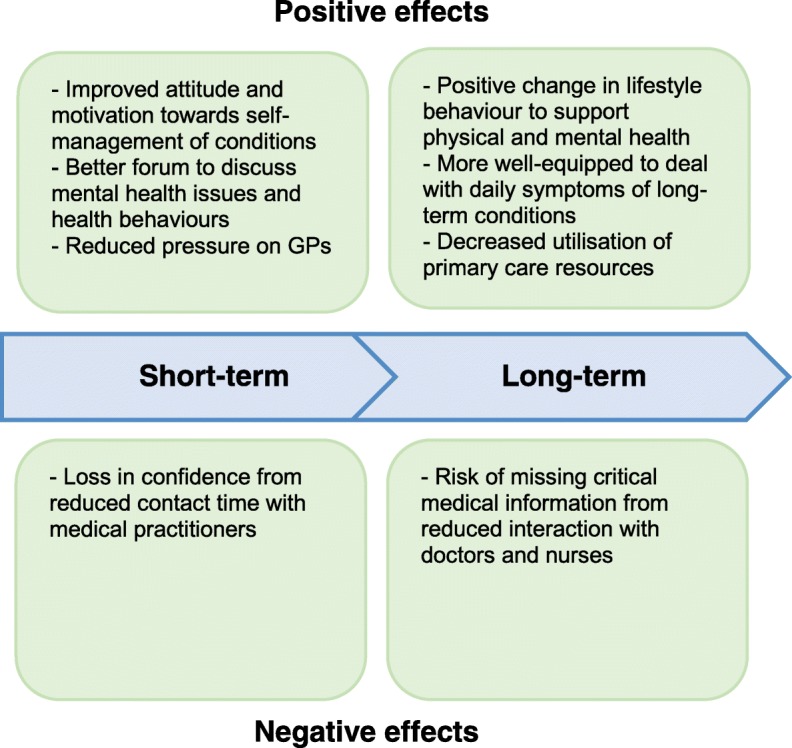
Logic model for Enhanced Primary Care showing our expectations of the short- and long-term effects on patients after the implementation of the intervention

Figure 1 presents a logic model for Enhanced Primary Care outlining our expectations of the short- and long-term effects of the intervention on patients based on our previous qualitative work and the existing health coaching literature.

## Methods

### Data

Our primary data source was the General Practice Patient Survey (GPPS) [37], which is an independent survey conducted annually since 2007 by research agency Ipsos MORI. The survey takes the form of a self-administered questionnaire and is sent at each wave by post to 2 million randomly selected patients from all GP practices in England. Eligible patients must be over 18 years of age, have an NHS number and be registered with a GP practice for the previous 6 months. Patients can receive the survey at multiple points in time but never in the same year. All responses are anonymous and individual patients cannot be tracked over time. The purpose of the survey is to gather information on patients’ experience of care and service received by their GP practices, and its annual nature enables changes in quality to be followed over time. We obtained survey data at the individual patient-level through NHS England.

### Intervention timing and controls

**Fig. 2:**
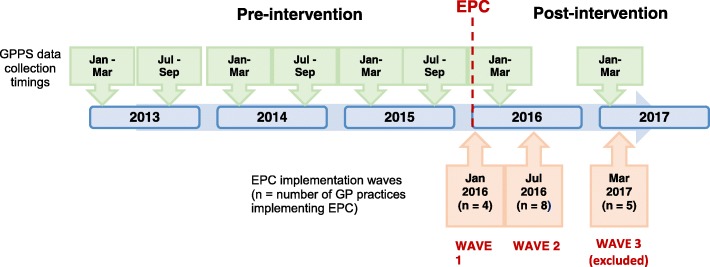
Timeline of analyses highlighting GP Practice Survey (GPPS) data collection timings and key dates of Enhanced Primary Care (EPC) implementation waves (number of GP practices implementing EPC in each wave). Time periods represent all pre- and post-intervention periods included in the analysis

Figure 2 summarises the timeline of analyses highlighting the pre- and post-intervention periods. Enhanced Primary Care was rolled out in three waves across 17 of the 19 GP practices in South Somerset. Two practices in South Somerset chose not to implement Enhanced Primary Care thus were excluded from the analysis. We were unable to exploit GPPS data collected after the implementation of the third wave due to removal of outcome measures from the survey, therefore we analysed only the first two waves (excluding the five practices implementing in wave 3). There are eight periods of data in total: six periods before and two periods after the implementation of the first Enhanced Primary Care wave (i.e. 14 months follow-up).

Table 2 presents our outcome measures for health status (EQ-5D-5L [38], physical functioning, psychological wellbeing and resilience), health behaviour (smoking habit), experience of care (person-centeredness and continuity of care), and health care (primary care) utilisation. Where an outcome is made up of multiple categorical measures, e.g. physical functioning, we allocated a score for each categorical measure and summed the scores to form a single overall score. See Additional file 1 for further details on the self-reported patient outcomes extracted from GPPS.

**Table 2:** Outcome measures extracted from GPPS

Outcomes	GPPS Measure	Description	Variable
*Health status*			
EQ-5D-5L	Mobility	Ability to walk	Continuous
	Self-care	Ability to dress and wash oneself	
	Usual activities	Performance in “work, study, housework, family or leisure activities”	
	Pain/discomfort	Level of pain and discomfort	
	Anxiety/depression	Level of anxiety and/or depression	
Physical functioning	Mobility		1 (lowest), 5 (highest) health status
	Usual activities		
	Pain/discomfort		
Psychological wellbeing	Anxiety/depression		1 (lowest), 5 (highest) health status
Resilience	Self-care		1 (lowest), 5 (highest) health status
	Confidence	Confidence in managing own health	
*Experience of care*			
Person-centeredness	GP rating	Rating of GP quality based on last appointment	1 (lowest), 5 (highest) quality
Continuity of care	Seeing preferred GP	Frequency of seeing preferred GP	1 (never or almost never), 2 (some of the time), 3 (a lot of the time), 4 (always or almost always)
*Health behaviour*			
Smoking habit	Smoking habit		1 (never smoked), 2 (former smoker), 3 (occasional smoker), 4 (regular smoker)
*Health care utilisation*			
Primary care utilisation	GP visit	Time since last GP appointment	1 (more than 12 months ago), 2 (between 6 and 12 months), 3 (between 3 and 6 months), 4 (in the past 3 months)
	Nurse visit	Time since last nurse appointment	1 (more than 12 months ago), 2 (between 6 and 12 months), 3 (between 3 and 6 months), 4 (in the past 3 months)

### Estimation strategy

We used a quasi-experimental design, difference-in-differences, exploiting longitudinal data from treatment and control groups. Since Enhanced Primary Care was implemented in multiple stages across the region, we adapted the standard difference-in-differences approach to allow for this staggered implementation [39, 40], i.e. intervention practices act as controls until they are included in the intervention.

The difference-in-differences analysis compares changes in outcomes over time between the patients registered with GP practices that have implemented Enhanced Primary Care and control patients registered with GP practices in the rest of England. We excluded patients registered to practices in all other vanguard sites in England (we were supplied with a list of relevant practice codes by NHS England) which might be implementing a similar model of care [41]. Since our model allows for a gradual joining of GP practices over time, the difference-in-differences estimate is a weighted average of all possible two-group/two-period difference-in-differences estimates in the data.

We conducted the analysis at the individual patient-level using a linear regression model, adjusting for sample weights. We used a weighting strategy to ensure comparability of individuals based on observed characteristics (i.e. age, gender, ethnicity, employment status, GP practice size, and Index of Multiple Deprivation) [42]. We excluded 1,368 individuals (0.04% of total observations) for which there was missing data on any of these observed characteristics. We included fixed effects for each GP practice and time period, and a vector of covariates for age, gender, employment status and ethnicity, and additionally clustered the standard errors at the GP practice-level to account for serial correlation. See Additional file 2 for further details on the weighting strategy and Additional file 3 for the equations used in our estimations. The statistical software, Stata (StataCorp, 2015), was used throughout our analysis.

Since Enhanced Primary Care is primarily directed at multimorbid patients, we conducted our main analysis on respondents reporting at least two chronic conditions (measured using two or more of 15 self-reported conditions – see Additional file 4). We performed additional analysis on all patients to assess population-level (spillover) effects. Table 3 presents the average proportions of the five most common combinations of chronic diseases (two and three diseases) reported by multimorbid patients (both treated and controls) across all survey waves included in our analysis.

**Table 3:** Average proportions of the five most common combinations of chronic conditions reported across all GPPS survey waves

Enhanced Primary Care Patients			%	Control Patients			%
*Patients with two chronic conditions*			*100*	*Patients with two chronic conditions*			*100*
Arthritis or long-term joint problem	High blood pressure		10.1	Arthritis or long-term joint problem	High blood pressure		10.7
Another long-term condition	High blood pressure		8.7	Diabetes	High blood pressure		9.6
Diabetes	High blood pressure		8.0	Another long-term condition	High blood pressure		7.3
Arthritis or long-term joint problem	Long-term back problem		5.0	Arthritis or long-term joint problem	Long-term back problem		5.8
Angina or long-term heart problem	High blood pressure		4.0	Asthma or long-term chest problem	High blood pressure		4.4
*Patients with three chronic conditions*			*100*	*Patients with three chronic conditions*			*100*
Arthritis or long-term joint problem	High blood pressure	Long-term back problem	6.9	Arthritis or long-term joint problem	High blood pressure	Long-term back problem	7.4
Arthritis or long-term joint problem	High blood pressure	Diabetes	4.3	Arthritis or long-term joint problem	High blood pressure	Diabetes	5.0
Arthritis or long-term joint problem	High blood pressure	Another long-term condition	4.1	Arthritis or long-term joint problem	High blood pressure	Another long-term condition	3.5
Arthritis or long-term joint problem	High blood pressure	Angina or long-term heart problem	3.2	Arthritis or long-term joint problem	High blood pressure	Asthma or long-term chest problem	3.5
Arthritis or long-term joint problem	High blood pressure	Asthma or long-term chest problem	4.1	Arthritis or long-term joint problem	High blood pressure	Angina or long-term heart problem	3.2

The difference-in-differences estimate is unbiased only under the assumption that in the absence of the intervention (i.e. Enhanced Primary Care), the difference between the treatment and control groups would be constant over time [43]. The difference-in-differences model relies on a "parallel trends" assumption [44] to ensure internal validity, and this was tested graphically and statistically (see Additional file 5).

For all estimates, a negative value indicates declining health status and care experience, a reduction in a detrimental health behaviour and decreasing care utilisation; a positive value indicates the opposite effects.

### Robustness checks

We included a range of robustness checks using alternative study designs to confirm our findings.

Robustness check 1 emulates the primary analysis but substitutes a control group composed of only NHS RightCare peers (see Additional file 6) [45]. NHS RightCare is a matching tool that generates the ten most similar Clinical Commissioning Groups (CCGs) for any given CCG based on a range of characteristics. With a tailored control group relevant to South Somerset, we expect these treatment effects to be the most comparative to the primary analysis.

Robustness check 2 conducts a two-group/two-period difference-in-differences analysis using GP practices that implemented Enhanced Primary Care in wave 1 only (i.e. excluding from the analysis the wave 2 treated practices). Wave 1 practices are those that have been the most exposed to the intervention, and so treatment effects ought to represent more pronounced medium-term effects. However, this analysis will be the most prone to any selection bias that occurred for early-uptake of the intervention.

Robustness check 3 conducts a two-group/two-period difference-in-differences analysis using all treated GP practices assuming they all join the treatment at a single point in time. This has the advantage of relaxing some of the additional assumptions of the staged adoption model, and minimising any selection bias from practices that implemented the intervention in later waves. However, the treatment effects will be significantly diluted since we are effectively treating a number of control practices as treated practices for at least one wave.

## Results

### Sample characteristics

Table 4 shows the standardised mean difference in outcome and control variables for treated and control respondents with multimorbidity in the pre-intervention period [46]. On average, those living in South Somerset are better off in terms of each of our outcome variables, compared to those living in the rest of England. A higher proportion are female, white, over 65, and fully retired from work in the treatment group (we adjusted for these as controls in our models), and they last contacted their GP less recently than the control group. See Additional file 7 for population-level sample characteristics.

**Table 4:** Pre-intervention summary statistics for multimorbid respondents

Variable	Enhanced Primary Care			Controls			SMD
	N	Mean^a^	SD	N	Mean^a^	SD	
*Outcomes*							
EQ-5D-5L score	1,143	0.6671	0.2511	601,527	0.6191	0.2803	0.1713
Physical functioning	1,195	0.6954	0.2382	634,358	0.6611	0.2493	0.1377
Psychological wellbeing	1,190	0.8445	0.2131	637,174	0.8011	0.2516	0.1725
Resilience	1,207	0.8335	0.1812	641,951	0.8051	0.1994	0.1422
Person-centeredness	1,102	0.8829	0.1550	591,869	0.8580	0.1794	0.1390
Continuity of care	952	0.7598	0.3041	439,459	0.7228	0.3113	0.1187
Smoking habit	1,220	0.2369	0.2647	655,448	0.2624	0.3004	−0.0849
Primary care utilisation	1,238	0.8538	0.1842	662,676	0.8461	0.1888	0.0407
*Individual characteristics*							
Male	1,250	0.4448	0.4971	673,788	0.4474	0.4972	−0.0052
White	1,250	0.9712	0.1673	673,788	0.8852	0.3188	0.2699
Full-time paid work	1,250	0.1280	0.3342	673,788	0.1308	0.3372	−0.0083
Fully retired from work	1,250	0.5880	0.4924	673,788	0.5334	0.4989	0.1095
Age under 35	1,250	0.0200	0.1401	673,788	0.0241	0.1532	−0.0265
Age 65 and over	1,250	0.6952	0.4605	673,788	0.6253	0.4840	0.1444
Last contacted GP < 6 months ago	1,250	0.8720	0.3342	673,788	0.8720	0.3341	−0.0001
Last contacted GP > 6 months ago	1,250	0.1248	0.3306	673,788	0.1212	0.3264	0.0109

*SMD* standardised mean difference (Cohen’s d)

^a^Unweighted means. Outcome means for physical functioning, psychological wellbeing, resilience, person centeredness, continuity of care, smoking habit and primary care utilisation normalised to [0,1]. Higher values indicate better health status and experience of care, increased smoking habit, and higher primary care utilisation

Continuity of care has a lower N compared to other outcomes since many patients indicated they did not have a preferred GP

### Testing for parallel trends

For multimorbid respondents, our primary analysis, visual and statistical evidence indicated comparable pre-intervention trends for all outcomes (see Additional file 5). At the population-level, we did not reject the parallel trends for all outcomes except psychological wellbeing. As psychological wellbeing within the treatment group was already on a decreasing trend relative to the controls before the introduction of Enhanced Primary Care, the difference-in-differences analysis estimate for this secondary analysis may overstate any beneficial policy effect on this outcome measure.

### Difference-in-differences analysis results

Tables 5 and 6 present the difference-in-differences results for multimorbid respondents. We find only two statistically significant results in the primary analysis: a decline in psychological wellbeing (− 0.0174 95% CI − 0.0283 to − 0.0065), − 2%; and person-centeredness (− 0.0356; 95% CI − 0.0530 to − 0.0183), − 4%.

**Table 5:** Difference-in-differences estimates of effect of Enhanced Primary Care on the multimorbid population (primary analysis)

Primary Analysis										
Outcomes	Unadjusted means				Unadjusted difference-in-differences			N	Adjusted^a^ difference-in-differences (95% CI)	
	Treatment Pre/Post		Control Pre/Post		Simplified	Wave 1	Wave 2			
*Health status*										
EQ-5D-5L	0.6671	0.6702	0.6431	0.6410	0.0052	−0.0226	0.0199^‡‡^	874,075	−0.0263	(−0.0679 to 0.0152)
Physical functioning	0.6954	0.7147	0.6778	0.6809	0.0161	−0.0098	0.0203^‡‡^	919,785	−0.0048	(−0.0424 to 0.0328)
Psychological wellbeing	0.8445	0.8226	0.8225	0.8148	−0.0142	−0.0408	0.0187^‡^	924,974	−0.0174	(−0.0283 to -0.0065)^‡‡^
Resilience	0.8335	0.8396	0.8207	0.8178	0.0091	−0.0008	0.0147^‡‡^	931,207	−0.0132	(−0.0758 to 0.0493)
*Experience of care*										
Person-centeredness	0.8829	0.8738	0.8662	0.8650	-0.0080	−0.0149^‡‡^	−0.0140^‡‡^	854,635	−0.0356	(−0.0530 to -0.0183)^‡‡^
Continuity of care	0.7598	0.7176	0.7410	0.7091	-0.0103	−0.0112^‡‡^	−0.0218^‡‡^	622,963	−0.0749	(−0.2011 to 0.0513)
*Health behaviour*										
Smoking habit	0.2369	0.2188	0.2446	0.2386	-0.0120	−0.0089	−0.0126	952,453	−0.0077	(−0.0273 to 0.0120)
*Health care utilisation*										
Primary care utilisation^b^	0.8538	0.8534	0.8470	0.8428	0.0038	0.0041	−0.0063^‡‡^	959,800	−0.0368	(−0.1022 to 0.0285)

All unadjusted and adjusted values are weighted. Simplified unadjusted difference-in-differences assumes that all treated practices join the treatment at a single point in time. Wave 1 and 2 unadjusted difference-in-differences represent the isolated effects on treated practices that joined in each wave. Adjusted intervention difference-in-differences incorporates the gradual implementation of the treatment, and controls for covariates and practice and time fixed effects

^a^Adjusted for gender, age, ethnicity, employment status, number of chronic conditions, time since last GP appointment, and practice and time fixed effects

^b^Adjusted for gender, age, ethnicity, employment status, number of chronic conditions, and practice and time fixed effects

^‡^*p* < 0.05, ^‡‡^*p* < 0.01

**Table 6:** Difference-in-differences estimates of effect of Enhanced Primary Care on the multimorbid population (robustness checks)

	Robustness check 1			Robustness check 2			Robustness check 3		
	(Alternative control group)			(Medium-term effects)			(Simplified analysis)		
Outcomes	N	Adjusted^a^ difference-in-differences (95% CI)		N	Adjusted^a^ difference-in-differences (95% CI)		N	Adjusted^a^ difference-in-differences (95% CI)	
*Health status*									
EQ-5D-5L	81,924	−0.0266	(−0.0702 to 0.0171)	874,075	−0.0104	(−0.0550 to 0.0340)	874,075	0.0014	(−0.0158 to 0.0186)
Physical functioning	85,457	−0.0084	(−0.0460 to 0.0292)	919,785	0.0078	(−0.0425 to 0.0582)	919,785	0.0096	(−0.0076 to 0.0268)
Psychological wellbeing	85,923	−0.0150	(−0.0150 to 0.1124)	924,974	−0.0514	(−0.0705 to -0.0324)^‡‡^	924,974	−0.0090	(−0.0301 to 0.0121)
Resilience	86,295	−0.0166	(−0.0803 to 0.0470)	931,207	0.0161	(−0.0168 to 0.0489)	931,207	0.0062	(−0.0090 to 0.0214)
*Experience of care*									
Person-centeredness	78,939	−0.0331	(−0.0566 to -0.0100)^‡‡^	854,635	−0.0212	(−0.0534 to 0.0110)	854,635	−0.0068	(−0.0218 to 0.0082)
Continuity of care	62,872	−0.0742	(−0.2108 to 0.0624)	622,963	−0.0340	(−0.1042 to 0.0361)	622,963	−0.0141	(−0.0530 to 0.0248)
*Health behaviour*									
Smoking habit	87,912	−0.0022	(−0.0349 to 0.0304)	952,453	0.0220	(−0.0204 to 0.0644)	952,453	−0.0160	(−0.0344 to 0.0023)
*Health care utilisation*									
Primary care utilisation^b^	88,595	−0.0402	(−0.1028 to 0.0223)	959,800	0.0025	(−0.0244 to 0.0294)	959,800	0.0064	(−0.0070 to 0.0197)

^a^Adjusted for gender, age, ethnicity, employment status, number of chronic conditions, time since last GP appointment, and practice and time fixed effects

^b^Adjusted for gender, age, ethnicity, employment status, number of chronic conditions, and practice and time fixed effects

^‡^*p* < 0.05, ^‡‡^*p* < 0.01

Using the RightCare comparator areas as controls (Robustness check 1), the effect on psychological wellbeing has a similar estimate although it is no longer significant with the smaller statistical power. Similar to the primary analysis, we find a decline in person-centeredness (− 0.0331; 95% CI − 0.0566 to − 0.0100), − 4%.

Looking at medium-term effects (Robustness check 2), the detrimental effect on psychological wellbeing found in the primary analysis remains significant (− 0.0541; 95% CI − 0.0705 to − 0.0324), − 6%, but the effect on person-centeredness is no longer significant.

Using a two-group/two-period model (Robustness check 3), as expected, the effects are smaller and not statistically significant: psychological wellbeing (− 0.0090; 95% CI − 0.0301 to 0.0121), − 1%; person-centeredness (− 0.0068; 95% CI − 0.0218 to 0.0082), − 1%.

Tables 7 and 8 present the difference-in-differences results for all respondents (i.e. population-level effects). We find two statistically significant results for outcomes dissimilar to those in the analysis for multimorbid respondents: a reduction in primary care utilisation (− 0.0331; 95% CI − 0.0448 to − 0.0214), − 5%; and a slight increase in smoking behaviour (0.0088; 95% CI 0.0009 to 0.0167), + 4%.

**Table 7:** Difference-in-differences estimates of effect of Enhanced Primary Care on the population (primary analysis)

Primary Analysis										
Outcomes	Unadjusted means				Unadjusted difference-in-differences			N	Adjusted^a^ difference-in-differences (95% CI)	
	Treatment Pre/Post		Control Pre/Post		Simplified	Wave 1	Wave 2			
*Health status*										
EQ-5D-5L	0.8192	0.8139	0.8060	0.8036	−0.0030	−0.0054	0.0054^‡‡^	3,147,503	0.0004	(−0.0103 to 0.0111)
Physical functioning	0.8557	0.8571	0.8438	0.8455	−0.0004	−0.0029	0.0048^‡‡^	3,242,123	0.0044	(−0.0116 to 0.0204)
Psychological wellbeing	0.9013	0.8892	0.8888	0.8824	−0.0056	−0.0143	0.0073^‡‡^	3,267,482	−0.0034	(−0.0078 to 0.0011)
Resilience	0.8927	0.8929	0.8829	0.8817	0.0015	0.0022	0.0046^‡‡^	3,238,682	0.0038	(−0.0139 to 0.0214)
*Experience of care*										
Person-centeredness	0.8759	0.8723	0.8503	0.8523	−0.0056	−0.0055^‡‡^	0.0010^‡‡^	2,919,957	−0.0155	(−0.0494 to 0.0184)
Continuity of care	0.7465	0.7083	0.7037	0.6787	−0.0132	−0.0173^‡‡^	−0.0187^‡‡^	1,774,615	−0.0794	(−0.2058 to 0.0470)
*Health behaviour*										
Smoking habit	0.2113	0.2034	0.2204	0.2128	−0.0002	0.0030	0.0046^‡‡^	3,368,788	0.0088	(0.0009 to 0.0167)^‡^
*Health care utilisation*										
Primary care utilisation^b^	0.7347	0.7299	0.7366	0.7294	0.0023	−0.0165^‡^	0.0091	3,384,804	−0.0331	(−0.0448 to -0.0214)^‡‡^

All unadjusted and adjusted values are weighted. Simplified unadjusted difference-in-differences assumes that all treated practices join the treatment at a single point in time. Wave 1 and 2 unadjusted difference-in-differences represent the isolated effects on treated practices that joined in each wave. Adjusted intervention difference-in-differences incorporates the gradual implementation of the treatment, and controls for covariates and practice and time fixed effects

^a^Adjusted for gender, age, ethnicity, employment status, number of chronic conditions, time since last GP appointment, and practice and time fixed effects

^b^Adjusted for gender, age, ethnicity, employment status, number of chronic conditions, and practice and time fixed effects

^‡^*p* < 0.05, ^‡‡^*p* < 0.01

**Table 8:** Difference-in-differences estimates of effect of Enhanced Primary Care on the population (robustness checks)

	Robustness Check 1			Robustness Check 2			Robustness Check 3		
	(Alternative Control Group)			(Medium-Term Effects)			(Simplified Analysis)		
Outcomes	N	Adjusted^a^ difference-in-differences (95% CI)		N	Adjusted^a^ difference-in-differences (95% CI)		N	Adjusted^a^ difference-in-differences (95% CI)	
*Health status*									
EQ-5D-5L	294,272	0.0014	(−0.0125 to 0.0153)	3,147,503	−0.0019	(−0.0156 to 0.0118)	3,147,503	−0.0023	(−0.0104 to 0.0059)
Physical functioning	301,710	0.0049	(−0.0126 to 0.0224)	3,242,123	0.0050	(−0.0129 to 0.0230)	3,242,123	−0.0003	(−0.0083 to 0.0076)
Psychological wellbeing	303,519	−0.0020	(−0.0107 to 0.0066)	3,267,482	−0.0211	(−0.0351 to -0.0071)^‡‡^	3,267,482	−0.0036	(−0.0110 to 0.0038)
Resilience	301,024	0.0035	(−0.0151 to 0.0220)	3,238,682	0.0033	(−0.0096 to 0.0163)	3,238,682	0.0007	(−0.0040 to 0.0055)
*Experience of care*									
Person-centeredness	268,370	−0.0143	(−0.0515 to 0.0230)	2,919,957	−0.0169	(−0.0566 to 0.0228)	2,919,957	−0.0065	(−0.0192 to 0.0061)
Continuity of care	178,630	−0.0774	(−0.2051 to 0.0503)	1,774,615	−0.0594	(−0.1141 to -0.0047)^‡^	1,774,615	−0.0128	(−0.0410 to 0.0154)
*Health behaviour*									
Smoking habit	311,270	0.0113	(−0.0017 to 0.0244)	3,368,788	0.0000	(−0.0410 to 0.0409)	3,368,788	−0.0011	(−0.0152 to 0.0131)
*Health care utilisation*									
Primary care utilisation^b^	312,654	−0.0372	(−0.0565 to -0.0178)^‡‡^	3,384,804	−0.0196	(−0.0525 to 0.0132)	3,384,804	0.0013	(−0.0126 to 0.0153)

^a^Adjusted for gender, age, ethnicity, employment status, number of chronic conditions, time since last GP appointment, and practice and time fixed effects

^b^Adjusted for gender, age, ethnicity, employment status, number of chronic conditions, and practice and time fixed effects

^‡^*p* < 0.05, ^‡‡^*p* < 0.01

Using the RightCare comparator areas as controls (Robustness check 1), we find a similar reduction in primary care utilisation (− 0.0372; 95% CI − 0.0565 to − 0.0178), − 5%, but the increase in smoking habit is not robust.

In the medium-term (Robustness check 2), the significant effects from our primary analysis disappear, but we report a decline in psychological wellbeing, in line with the multimorbidity analysis (− 0.0211; 95% CI − 0.0351 to − 0.0071), − 2%; and a reduction in continuity of care (− 0.0594; 95 CI − 0.1141 to − 0.0047), − 8%.

Using the two-group/two-period simplified model (Robustness check 3), we find no significant effects.

## Discussion

Our results show that there is very little effect of health coaching on patient experiences or outcomes in the short-to-medium term. For multimorbid patients, we find evidence of small negative effects on psychological wellbeing (short and medium term) and person-centeredness (short term). At the population-level, we find slight reductions in primary care utilisation (short-term), and there might be a small negative effect on psychological wellbeing and continuity of care in the medium term. These findings are in line with our logic model and evidence from our previous qualitative work where some patients felt a sense of abandonment with the new model, i.e. “does my GP not want me anymore” [15], and the non-medical training of the health coaches might have been perceived as a negative. However, our qualitative evidence finds that the Symphony programme has identified significant unmet social and mental health needs. Therefore, they may partially represent an increase in previously undiagnosed anxiety or depression. At the population-level, in the short-term only, the intervention appears to promote a shift in chronic disease management away from medical practitioners towards health coaches, thus potentially enabling doctors and nurses to focus more time on acute patients.

### Results in context

The aim of health coaching is to empower patients to self-manage their chronic conditions through greater knowledge, skills and confidence. However the evidence for this approach is mixed [47]. On one hand, researchers have highlighted self-care as key to the management of chronic conditions [48], with findings of positive behavioural changes associated with increased patient activation [49, 50]. On the other hand, they have found a range of barriers to self-management including low health literacy and the challenging burden associated with chronic conditions (especially for patients with multiple conditions) [51–53]. Our results display similar mixed behaviour, in that certain variables tend to lose significance or change direction in the medium-term. For example, the negative effect on person-centeredness in the multimorbid analysis disappears. These results could be a reflection of the temporary challenges facing patients when transitioning from provider- to self-led care and may not be entirely representative of longer-term effects.

Health coaching can take a range of forms and is likely to differ in terms of time duration, and the health coaches’ professional background, medical qualifications and overall competency [24]. For this reason, implementing a health coaching initiative based on existing evidence can prove challenging, as indicated by the results. The Enhanced Primary Care model is based on a similar scheme implemented in the US, where most of the health coaching literature originates. While the US model may provide a valuable insight into the framework of the initiative, it is important to highlight the vast differences between the UK and US health care systems, predominantly in baseline strength of primary care, and how this may translate to differences in our core outcomes [26].

### Policy implications

Our study offers important contributions to the existing literature on primary care workforce development and task-shifting, most notably relevant to the implementation of the NHS Long Term Plan [10]. First, we highlight that workforce diversification achieved through the substitution of medical for non-medical practitioners may produce undesirable results, particularly in the short-term as patients adjust to the ‘up-skilling’. Second, we find that prevention-based strategies, particularly for multimorbid patients, are likely to have only limited (if any) beneficial impact in the short-term, even on aspects of patient experience and health behaviours which we might expect to be more influenceable in this time horizon.

The negative effect we find on psychological wellbeing is an interesting finding given the NHS’, and global health system, commitments to strengthening care for mental health [54, 55]. A large proportion of patients with long-term physical health conditions frequently experience co-morbid mental health problems [56]. GPs often struggle to allocate consultation time for these co-morbidities [57, 58], therefore health coaches aim to fill this gap by offering patients a platform to discuss their mental health [21]. This strategy of substituting primary care away from medically-qualified professionals towards those less-qualified has become a growing area of interest in recent years. Previous studies have evaluated the substitution of nurses for doctors in primary care to counter rising demand pressures, finding that for the management of chronic conditions and ongoing care, nurses can achieve equal or even better health outcomes and quality of care compared to GPs [59, 60]. In particular, nurse-led self-management support of patients with diabetes and cardiovascular disease appears to be more effective than physician-led approaches in reducing blood pressure and blood glucose levels [61]. Additional research conducted in the US has emphasised the estimated savings in doctor time that could be achieved through greater reallocation of chronic disease management to health coaches [62]. However, the fact that, unlike the US, UK health coaches do not tend to be medically trained, may help to explain the decline in each of the indicators for health status in multimorbid patients (as above, perhaps only caused by the perception of the patient rather than necessarily the skills of the health coach).

### Strengths and weaknesses of the study

We used a robust difference-in-differences analysis that satisfied the parallel trends assumption [63]. We applied several robustness checks by varying the study design and the composition of the control group. The model enabled the evaluation of a regional-level intervention using data from routine practice, with scope for external validity and generalisability of the results to other regions in England. Our findings are plausible and are supported by the logic model, our parallel qualitative research [15], and previous literature on health coaching [49, 50].

However, our study is subject to a number of weaknesses. The Complex Care Hub was implemented alongside Enhanced Primary Care, and since both initiatives target similar objectives relating to integrated care, there is the possibility of capturing a combined effect. We acknowledged this limitation by using a multiple-start difference-in-differences model that fully incorporates the timing structure of Enhanced Primary Care.

We cannot identify the specific patients that were exposed to the intervention in our dataset, meaning any direct effect might be diluted. However, we focussed our primary analysis on those with multimorbidity; those with more probability of being directly treated. Furthermore, we recognise that Enhanced Primary Care is a population health-level model that, through an overall restructuring of the primary care service, intends to improve outcomes for the entire population. This is likely to include spillover effects which our analysis method was able to capture.

The dataset we used is repeated cross-sections of the national patient experience across all GP practices. However, chronic conditions are self-reported, so we do not have an indication of severity. Also, we were not able to track patients over time. This limited our ability to conduct in-depth subgroup analyses, for example patients with specific combinations of diseases, as we might risk concerns of a ‘bad control’ [39] since we are unable to fix the combination of diseases for an individual in the pre-intervention period.

### Future research

Given that we could only include a post-intervention time period of 14 months, further work is required to confirm our initial short and medium-term results, and to extend the analysis beyond this time period to measure the longer-term effects. Future research could also include additional measures of health behaviour (e.g. alcohol intake, diet, level of exercise), where available, as outcome variables. Our multimorbidity dummy was constructed through a single count of chronic conditions presented in the GPPS. However, the number of conditions is not in itself an indicator of overall need of care (some multimorbid patients may require more intensive care than others). Further work could explore heterogeneous effects on different samples of multimorbid patients to understand which “types” might benefit most from the intervention. The measure of care utilisation could be extended to primary and secondary care costs, with an economic evaluation exploring whether health coaching positively impacts cost per quality adjusted life-year compared to usual care.

## Conclusion

We show that Enhanced Primary Care does not achieve its primary objective of improving patient experience and outcomes in the short-to-medium term (up to 14 months). Our most consistent finding is slightly lower psychological wellbeing and person-centeredness amongst multimorbid patients (it might be initially difficult for patients to adjust to the model). We do, however, show potentially positive primary care utilisation effects in terms of reducing demand and time pressures on medical practitioners, although this is not a lasting effect. The results raise timely evidence regarding suitable primary care workforce changes advocated in the NHS Long Term Plan, and the time horizon of any benefits of prevention strategies, thus highlighting the need for further research into the long-term effects of health coaching.

## Additional files

Additional file 1: Outcome measures. (DOCX 15 kb)

Additional file 2: Weighting strategy. (DOCX 18 kb)

Additional file 3: Difference-in-Differences analysis. (DOCX 16 kb)

Additional file 4: Multimorbidity measure. (DOCX 13 kb)

Additional file 5: Parallel trends assumption. (DOCX 125 kb)

Additional file 6: Robustness checks. (DOCX 130 kb)

Additional file 7: Population-level sample characteristics. (DOCX 19 kb)

## Data Availability

The data that support the findings of this study are available from Ipsos Mori (on behalf of NHS England) but restrictions apply to the availability of these data, which were used under license for the current study, and so are not publicly available. Data are however available from the authors upon reasonable request and with permission of Ipsos Mori.

## Abbreviations

CCG: Clinical Commissioning Group
EPC: Enhanced Primary Care
GP: General Practice
GPPS: General Practice Patient Survey
