# Supplementary material for: Effects of non-medical health coaching on multimorbid patients in primary care: a difference-in-differences analysis
Source: BMC Health Serv Res. 2019 Aug 22;19:593. doi: 10.1186/s12913-019-4367-8 (PMC6704561; doi:10.1186/s12913-019-4367-8)
Supplement: Supplementary file 1 — Outcome measures. (DOCX 15 kb) [file 12913_2019_4367_MOESM1_ESM.docx]

# **Additional file 1**

# **Outcome measures**

Outcome measures were constructed using the following questions:

[The number in brackets denotes the score attached to each answer]

**1. Please indicate which statements best describe your own health state today**

*Mobility*

□ I have no problems in walking about (5)

□ I have slight problems in walking about (4)

□ I have moderate problems in walking about (3)

□ I have severe problems in walking about (2)

□ I am unable to walk about (1)

*Self-Care*

□ I have no problems washing or dressing myself (5)

□ I have slight problems washing or dressing myself (4)

□ I have moderate problems washing or dressing myself (3)

□ I have severe problems washing or dressing myself (2)

□ I am unable to wash or dress myself (1)

*Usual Activities (e.g. work, study, housework, family or leisure activities)*

□ I have no problems doing my usual activities (5)

□ I have slight problems doing my usual activities (4)

□ I have moderate problems doing my usual activities (3)

□ I have severe problems doing my usual activities (2)

□ I am unable to do my usual activities (1)

*Pain / Discomfort*

□ I have no pain or discomfort (5)

□ I have slight pain or discomfort (4)

□ I have moderate pain or discomfort (3)

□ I have severe pain or discomfort (2)

□ I have extreme pain or discomfort (1)

*Anxiety / Depression*

□ I am not anxious or depressed (5)

□ I am slightly anxious or depressed (4)

□ I am moderately anxious or depressed (3)

□ I am severely anxious or depressed (2)

□ I am extremely anxious or depressed (1)

**2. How confident are you that you can manage your own health?**
□ Very confident (4)

□ Fairly confident (3)

□ Not very confident (2)

□ Not at all confident (1)

**3. Last time you saw or spoke to a GP from your GP surgery, how good was that GP at each of the following?**

*Giving you enough time
Listening to you
Explaining tests and treatments
Involving you in decisions about your care
Treating you with care and concern*

□ Very good (5)

□ Good (4)

□ Neither good nor poor (3)

□ Poor (2)

□ Very poor (1)

□ Doesn’t apply

**4a. Is there a particular GP you usually prefer to see or speak to?**

□ Yes (see part b)

□ No

**4b. How often do you see or speak to the GP you prefer?**

□ Always or almost always (4)

□ A lot of the time (3)

□ Some of the time (2)

□ Never or almost never (1)

□ Not tried at this GP surgery

**5. Which of the following best describes your smoking habits?**

□ Never smoked (1)

□ Former smoker (2)

□ Occasional smoker (3)

□ Regular smoker (4)

**6. When did you last see or speak to a GP from your GP surgery?**

□ In the past 3 months (5)

□ Between 3 and 6 months ago (4)

□ Between 6 and 12 months ago (3)

□ More than 12 months ago (2)

□ I have never seen a GP from my GP surgery (1)

**7. When did you last see or speak to a nurse from your GP surgery?**

□ In the past 3 months (5)

□ Between 3 and 6 months ago (4)

□ Between 6 and 12 months ago (3)

□ More than 12 months ago (2)

□ I have never seen a nurse from my GP surgery (1)
