# Supplementary material for: Effects of non-medical health coaching on multimorbid patients in primary care: a difference-in-differences analysis
Source: BMC Health Serv Res. 2019 Aug 22;19:593. doi: 10.1186/s12913-019-4367-8 (PMC6704561; doi:10.1186/s12913-019-4367-8)
Supplement: Supplementary file 2 — Weighting strategy. (DOCX 18 kb) [file 12913_2019_4367_MOESM2_ESM.docx]

# **Additional file 2**

# **Weighting strategy**

The weighting strategy used in this study is based on the work by Stuart et al (2014)^[[1]](#footnote-1)^ describing the use of propensity scores in conjunction with difference-in-differences models in policy impact evaluation.

All regression models were weighted to ensure similarity between observations on selected observed characteristics. The weighting strategy used in this study is suitable for data from repeated cross-sections by enabling “Groups” to be weighted according to both treatment status (treated/control) and time (pre/post). The four Groups (treated pre, treated post, control pre, control post) were weighted to be balanced on a set of characteristics that reflect the covariate distribution of the treated group during the pre-period.

The variable, Group, is defined as follows:

$$Group z= \left\{ \begin{aligned} 1 if y=1, t=0 \\ 2 if y=1, t=1 \\ 3 if y=0, t=0 \\ 4 if y=0, t=1 \end{aligned} \right.$$

where $y=$ treatment status (=0 if control, =1 if treated), $, t=$ time period (=0 if pre-EPC, =1 if post-EPC).

The weights were constructed using the propensity score, which represents the probability of being in Group 1 versus otherwise (Group 2, 3 or 4). The propensity score was estimated using a multinomial logistic regression predicting Group as a function of certain observed characteristics ($X_{i}$) including age, gender, ethnicity, employment status, GP practice size (categorised by decile) and IMD (Index of Multiple Deprivation) (categorised by decile). Individuals with missing data on any of the observed characteristics were eliminated from the analysis (i.e. 1,368 individuals, 0.04% of total individuals). STATA’s ‘pscore’ command is used to calculate the propensity score.^[[2]](#footnote-2)^

There were four resulting propensity scores per patient $i$:

$$e_{z}\left( X_{i} \right); z=1, 2, 3, 4$$

The final weights were calculated so that the propensity scores for each group that patient $i$ was actually in ($g)$ are weighted against Group 1:

$w_{i}=\frac{e_{1}\left( X_{i} \right)}{e_{g}}$; $g=1, 2, 3, 4$

By fitting a weighted regression model using weights $w_{i}$, the resulting difference-in-differences analysis estimates are consistent even if there exists selection bias due to confounding issues across the four groups.

1. Stuart EA, Huskamp HA, Duckworth K, Simmons J, Song Z, Chernew ME, et al. Using propensity scores in difference-in-differences models to estimate the effects of a policy change. Health Services and Outcomes Research Methodology. 2014;14(4):166-82. [↑](#footnote-ref-1)
2. Becker SO, Ichino A. Estimation of Average Treatment Effects Based on Propensity Scores. The Stata Journal. 2002;2(4):358-77. [↑](#footnote-ref-2)
