# Supplementary material for: Effects of non-medical health coaching on multimorbid patients in primary care: a difference-in-differences analysis
Source: BMC Health Serv Res. 2019 Aug 22;19:593. doi: 10.1186/s12913-019-4367-8 (PMC6704561; doi:10.1186/s12913-019-4367-8)
Supplement: Supplementary file 3 — Difference-in-Differences analysis. (DOCX 16 kb) [file 12913_2019_4367_MOESM3_ESM.docx]

# **Additional file 3**

# **Difference-in-Differences analysis**

# The difference-in-differences model with staged implementation uses a time fixed effect, instead of the conventional dummy variable, to indicate the post-treatment period. As the model allows for a gradual joining of GP practices over time, the difference-in-differences estimate is a weighted average of all possible two-group/two-period difference-in-differences estimates in the data.^[[1]](#footnote-1)^

# The equation used in our analysis takes the following form:

$$y_{it}= \beta_{1} {EPC}_{it}+ \delta_{t}+ \boldsymbol{xk}_{it}+ \alpha_{j}+ \epsilon_{it}$$

Where:

$y_{it}=$ outcome of person i in time t

${EPC}_{it}$ = dummy for treatment status

$\delta_{t}$ = time fixed-effects

$\boldsymbol{xk}_{it}$ = vector of individual covariates (age category, gender, ethnicity, employment status, and indicator variables for chronic health condition and time since last GP visit)

$\alpha_{j}$ = constant with absorbed practice fixed-effects

$\epsilon_{it}$ = random error

The estimate of Enhanced Primary Care on patients is the coefficient, $\beta_{1}$.

1. Goodman-Bacon A. Difference-in-Differences with Variation in Treatment Timing. National Bureau of Economic Research Working Paper Series. 2018;25018 [↑](#footnote-ref-1)
