# Supplementary material for: Effects of non-medical health coaching on multimorbid patients in primary care: a difference-in-differences analysis
Source: BMC Health Serv Res. 2019 Aug 22;19:593. doi: 10.1186/s12913-019-4367-8 (PMC6704561; doi:10.1186/s12913-019-4367-8)
Supplement: Supplementary file 4 — Multimorbidity measure. (DOCX 13 kb) [file 12913_2019_4367_MOESM4_ESM.docx]

# **Additional file 4**

# **Multimorbidity measure**

Multimorbid patients are classified as having at least two of the following medical conditions:

Alzheimer’s disease or dementia

Angina or long-term heart problem

Arthritis or long-term joint problem

Asthma or long-term chest problem

Blindness or severe visual impairment

Cancer in the last 5 years

Deafness or severe hearing impairment

Diabetes

Epilepsy

High blood pressure

Kidney or liver disease

Learning difficulty

Long-term back problem

Long-term mental health problem

Long-term neurological problem

Another long-term condition
