# Supplementary material for: Effects of non-medical health coaching on multimorbid patients in primary care: a difference-in-differences analysis
Source: BMC Health Serv Res. 2019 Aug 22;19:593. doi: 10.1186/s12913-019-4367-8 (PMC6704561; doi:10.1186/s12913-019-4367-8)
Supplement: Supplementary file 5 — Parallel trends assumption. (DOCX 125 kb) [file 12913_2019_4367_MOESM5_ESM.docx]

# **Additional file 5**

# **Parallel trends assumption**

*Patient-level average trends using overlay graphs: treated (‘Treated’) versus control (‘Control’), treated with multimorbidity (‘TreatedMM’) versus control with multimorbidity (‘ControlMM’).
[Vertical line represents EPC wave 1 implementation. Time corresponds to GPPS data collection timings (2 = Jan-Mar 2013, 3 = Jul-Sep 2013, 4 = Jan-Mar 2014, 5 = Jul-Sep 2014, 6 = Jan-Mar 2015, 7 = Jul-Sep 2016, 8 = Jan-Mar 2016, 9 = Jan-Mar 2017)]*

EQ-5D-5L Physical functioning


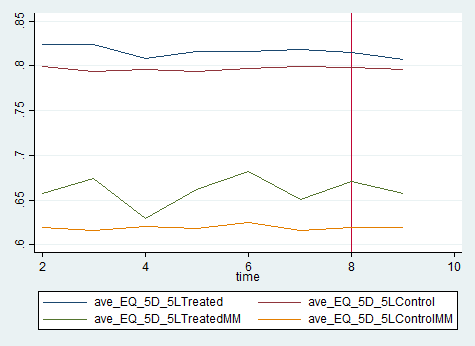

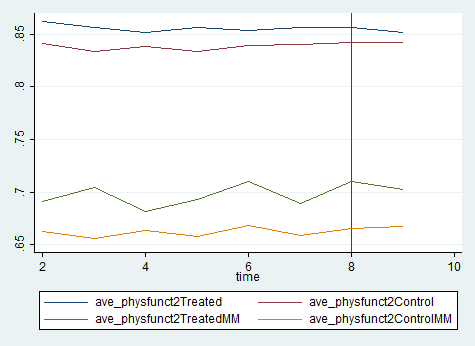


Psychological wellbeing Resilience


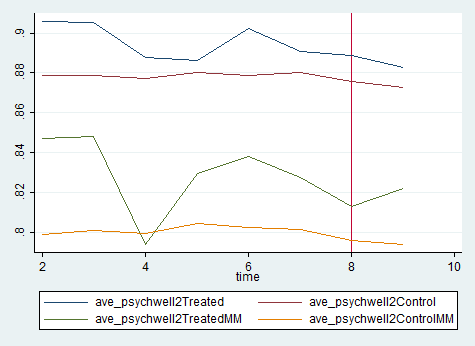

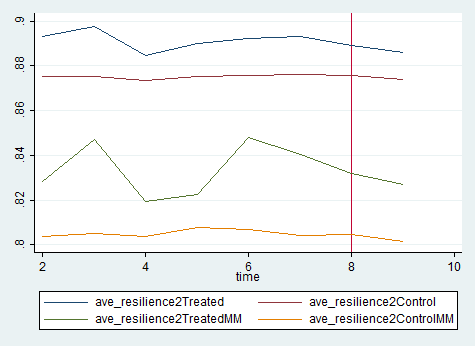


Person centeredness Continuity of care


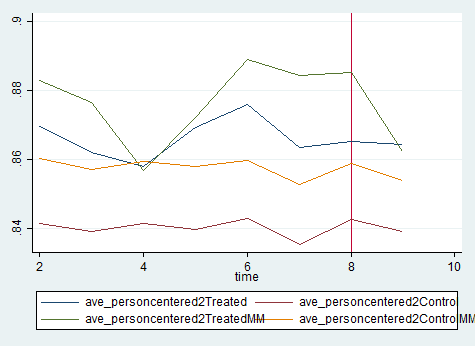

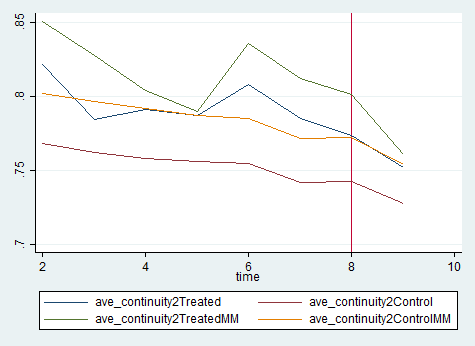


Smoking habit Primary care utilisation


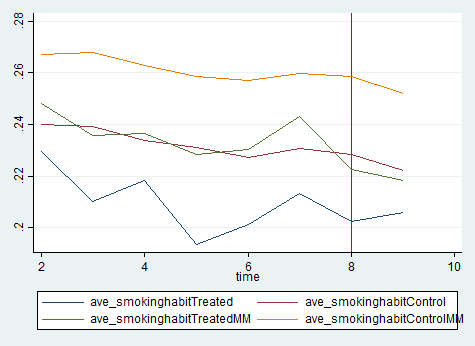

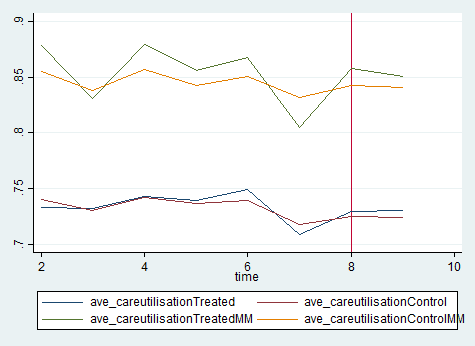


The statistical test uses an interaction between the continuous linear time trend and the treatment dummy using pre-intervention weighted data only, and controls for covariates and fixed effects. An F-test was used to assess statistical significance.

| Statistical F-test for parallel trends for multimorbid respondents | | | | |
| --- | --- | --- | --- | --- |
| **Variable** | **N** | **F-statistic** | **p-value** |  |
|  |  |  |  |  |
| *Outcomes* |  |  |  |  |
| EQ-5D-5L | 602,670 | 0.00 | 0.7670 |  |
| Physical functioning | 635,553 | 0.00 | 0.9597 |  |
| Psychological wellbeing | 638,364 | 0.25 | 0.6159 |  |
| Resilience | 643,158 | 1.52 | 0.2177 |  |
| Person-centeredness | 592,971 | 1.14 | 0.2853 |  |
| Continuity of care | 441,671 | 0.29 | 0.5912 |  |
| Smoking habit | 656,668 | 0.01 | 0.9260 |  |
| Primary care utilisation | 663,914 | 3.18 | 0.0748 |  |
| Adjusted for gender, age, ethnicity, employment status, number of chronic conditions, time since last GP appointment (except for primary care utilisation), and practice and time fixed effects. ^‡^p<0.05, ^‡‡^p<0.01 | | | | |
|  |  |  |  |  |
|  |  |  |  |  |

| Statistical F-test for parallel trends for all respondents | | | |  |
| --- | --- | --- | --- | --- |
| **Variable** | **N** | **F-statistic** | **p-value** |  |
|  |  |  |  |  |
| *Outcomes* |  |  |  |  |
| EQ-5D-5L | 2,153,181 | 0.30 | 0.5827 |  |
| Physical functioning | 2,220,502 | 0.24 | 0.6256 |  |
| Psychological wellbeing | 2,236,524 | 10.62 | 0.0011^‡‡^ |  |
| Resilience | 2,216,395 | 0.16 | 0.6881 |  |
| Person-centeredness | 2,012,401 | 0.1 | 0.7523 |  |
| Continuity of care | 1,265,963 | 0.1 | 0.7485 |  |
| Smoking habit | 2,307,873 | 0.6 | 0.4378 |  |
| Primary care utilisation | 2,324,497 | 0.33 | 0.5634 |  |
| Adjusted for gender, age, ethnicity, employment status, number of chronic conditions, time since last GP appointment (except for primary care utilisation), and practice and time fixed effects. ^‡^p<0.05, ^‡‡^p<0.01 | | | | |
|  |  |  |  |  |
|  |  |  |  |  |
