# Supplementary material for: Effects of non-medical health coaching on multimorbid patients in primary care: a difference-in-differences analysis
Source: BMC Health Serv Res. 2019 Aug 22;19:593. doi: 10.1186/s12913-019-4367-8 (PMC6704561; doi:10.1186/s12913-019-4367-8)
Supplement: Supplementary file 6 — Robustness checks. (DOCX 130 kb) [file 12913_2019_4367_MOESM6_ESM.docx]

# **Additional file 6**

# **Robustness checks**

**Robustness check 1**

We used the “Similar 10 CCG Explorer Tool” to identify South Somerset’s NHS RightCare peers. As South Somerset falls within the broader NHS Somerset Clinical Commissioning Group (CCG), we used NHS Somerset as our input CCG. This tool creates a bespoke group of 10 similar CCGs using weightings of 12 variables: overall deprivation, health deprivation, population total, population aged under 5 years, population aged 5-14 years, population aged 15-24 years, population aged 75+ years, ADSONS, population density, population density slope, percentage of black ethnicity and percentage of Asian ethnicity. Further information can be found here: <https://www.england.nhs.uk/publication/similar-10-ccg-explorer-tool/>

The tool generated the following 10 CCGs as NHS Somerset’s RightCare peers: NHS Kernow CCG, NHS Gloucestershire CCG, NHS Ipswich and East Suffolk CCG, NHS Wiltshire CCG, NHS West Hampshire CCG, NHS Coastal West Sussex CCG, NHS South Worcestershire CCG, NHS Dorset CCG, NHS East Riding of Yorkshire CCG, NHS Cumbria CCG. The control group in this analysis thus comprised only of individuals living in these 10 CCGs.

Parallel trends

*Patient-level average trends using overlay graphs: treated (‘Treated’) versus control (‘Control’), treated with multimorbidity (‘TreatedMM’) versus control with multimorbidity (‘ControlMM’). [Vertical line represents EPC wave 1 implementation. Time corresponds to GPPS data collection timings (2 = Jan-Mar 2013, 3 = Jul-Sep 2013, 4 = Jan-Mar 2014, 5 = Jul-Sep 2014, 6 = Jan-Mar 2015, 7 = Jul-Sep 2016, 8 = Jan-Mar 2016, 9 = Jan-Mar 2017)]*

EQ-5D-5L Physical functioning


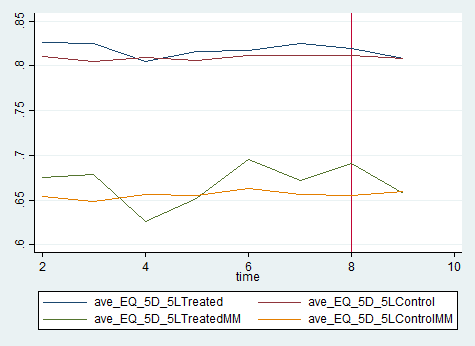

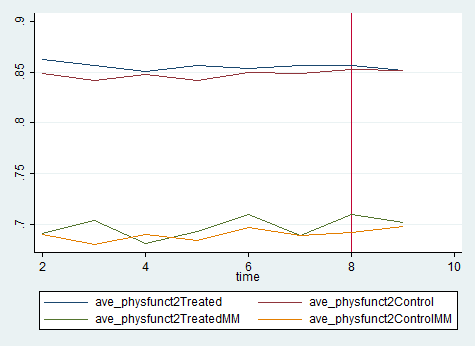


Psychological wellbeing Resilience


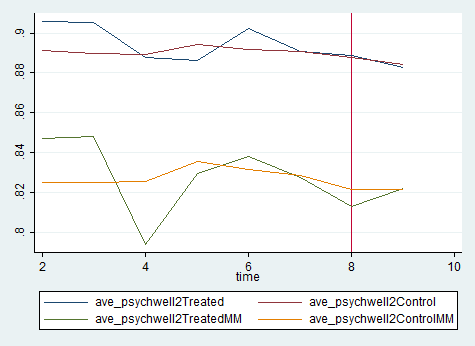

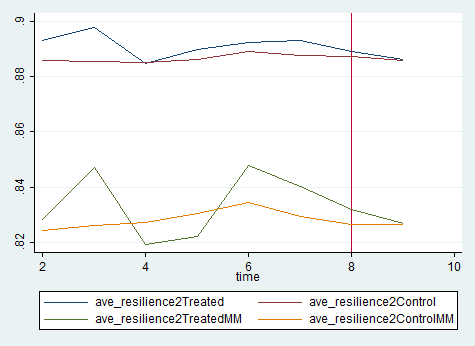


Person centeredness Continuity of care


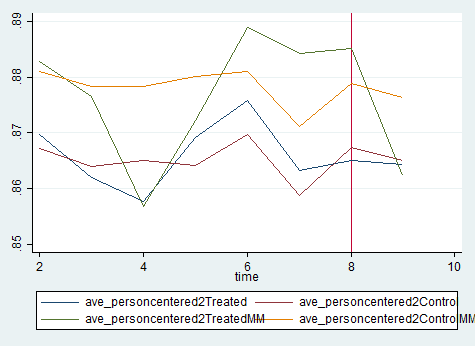

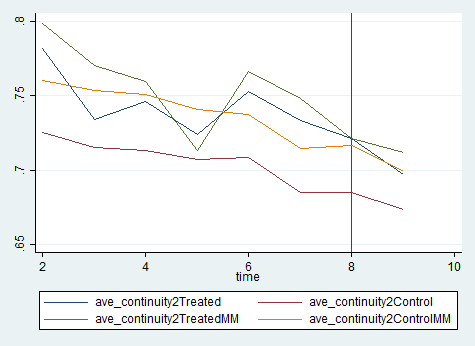


Smoking habit Primary care utilisation


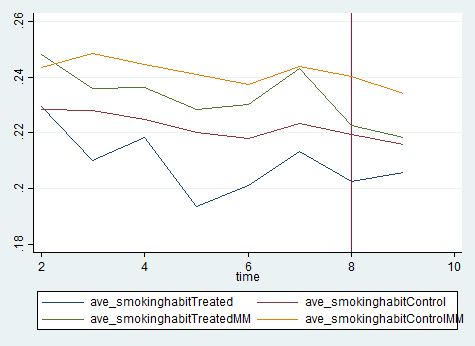

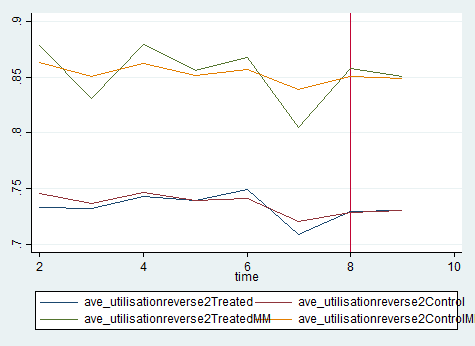


| Statistical F-test for parallel trends for multimorbid respondents | | | | |
| --- | --- | --- | --- | --- |
| **Variable** | **N** | **F-statistic** | **p-value** |  |
|  |  |  |  |  |
| *Outcomes* |  |  |  |  |
| EQ-5D-5L | 56,540 | 0.02 | 0.8809 |  |
| Physical functioning | 59,079 | 0.03 | 0.8592 |  |
| Psychological wellbeing | 59,300 | 0.11 | 0.7351 |  |
| Resilience | 59,584 | 0.64 | 0.4223 |  |
| Person-centeredness | 54,651 | 1.07 | 0.3025 |  |
| Continuity of care | 44,474 | 0.51 | 0.4734 |  |
| Smoking habit | 60,601 | 0.05 | 0.8263 |  |
| Primary care utilisation | 61,223 | 2.78 | 0.0962 |  |
| Adjusted for gender, age, ethnicity, employment status, number of chronic conditions, time since last GP appointment (except for primary care utilisation), and practice and time fixed effects. ^‡^p<0.05, ^‡‡^p<0.01 | | | | |
|  |  |  |  |  |
|  |  |  |  |  |

| Statistical F-test for parallel trends for all respondents | | | |  |
| --- | --- | --- | --- | --- |
| **Variable** | **N** | **F-statistic** | **p-value** |  |
|  |  |  |  |  |
| *Outcomes* |  |  |  |  |
| EQ-5D-5L | 201,082 | 1.64 | 0.2007 |  |
| Physical functioning | 206,380 | 1.17 | 0.2802 |  |
| Psychological wellbeing | 207,451 | 10.49 | 0.0013^‡‡^ |  |
| Resilience | 205,728 | 1.13 | 0.2891 |  |
| Person-centeredness | 184,445 | 0.00 | 0.9639 |  |
| Continuity of care | 126,661 | 0.24 | 0.6278 |  |
| Smoking habit | 212,812 | 2.99 | 0.0842 |  |
| Primary care utilisation | 214,222 | 4.08 | 0.0439^‡^ |  |
| Adjusted for gender, age, ethnicity, employment status, number of chronic conditions, time since last GP appointment (except for primary care utilisation), and practice and time fixed effects. ^‡^p<0.05, ^‡‡^p<0.01 | | | | |
|  |  |  |  |  |
|  |  |  |  |  |

**Robustness check 2**

$$y_{it}= \beta_{1} {EPC}_{i}+ \beta_{2} {PostEPC}_{t}+\beta_{3} {EPC}_{i} \times{PostEPC}_{t}+ \delta_{t}+ \boldsymbol{xk}_{it}+ \alpha_{j}+ \epsilon_{it}$$

Where:

${PostEPC}_{t}$ = dummy for time periods post-EPC

The estimate of Enhanced Primary Care on patients is the coefficient, $\beta_{3}$

**Robustness check 3**

This model takes the same form as robustness check 2.
