# Supplementary material for: Effects of non-medical health coaching on multimorbid patients in primary care: a difference-in-differences analysis
Source: BMC Health Serv Res. 2019 Aug 22;19:593. doi: 10.1186/s12913-019-4367-8 (PMC6704561; doi:10.1186/s12913-019-4367-8)
Supplement: Supplementary file 7 — Population-level sample characteristics. (DOCX 19 kb) [file 12913_2019_4367_MOESM7_ESM.docx]

# **Additional file 7**

# **Population-level sample characteristics**

|  |  | **Enhanced Primary Care** | | |  | **Controls** | | |  | **SMD** |
| --- | --- | --- | --- | --- | --- | --- | --- | --- | --- | --- |
| **Variable** |  | **N** | **Mean*** | **SD** |  | **N** | **Mean*** | **SD** |  |  |
| *Outcomes* |  |  |  |  |  |  |  |  |  |  |
| EQ-5D-5L score |  | 4,202 | 0.8192 | 0.2081 |  | 2,148,979 | 0.7967 | 0.2366 |  | 0.0951 |
| Physical functioning |  | 4,308 | 0.8557 | 0.1966 |  | 2,216,194 | 0.8374 | 0.2155 |  | 0.0848 |
| Psychological wellbeing |  | 4,307 | 0.9013 | 0.1771 |  | 2,232,217 | 0.8789 | 0.2065 |  | 0.1082 |
| Resilience |  | 4,278 | 0.8927 | 0.1385 |  | 2,212,117 | 0.8752 | 0.1517 |  | 0.1153 |
| Person-centeredness |  | 3,818 | 0.8759 | 0.1582 |  | 2,008,583 | 0.8400 | 0.1859 |  | 0.1929 |
| Continuity of care |  | 2,821 | 0.7465 | 0.3069 |  | 1,256,102 | 0.6824 | 0.3241 |  | 0.1981 |
| Smoking habit |  | 4,418 | 0.2113 | 0.2751 |  | 2,303,455 | 0.2339 | 0.3080 |  | -0.0736 |
| Primary care utilisation |  | 4,454 | 0.7347 | 0.2548 |  | 2,320,043 | 0.7350 | 0.2536 |  | -0.0011 |
|  |  |  |  |  |  |  |  |  |  |  |
| *Individual characteristics* |  |  |  |  |  |  |  |  |  |  |
| Male |  | 4,504 | 0.4218 | 0.4939 |  | 2,356,547 | 0.4265 | 0.4946 |  | -0.0094 |
| White |  | 4,504 | 0.9707 | 0.1687 |  | 2,356,547 | 0.8524 | 0.3547 |  | 0.3337 |
| Full-time paid work |  | 4,504 | 0.2875 | 0.4527 |  | 2,356,547 | 0.3214 | 0.4670 |  | -0.0725 |
| Fully retired from work |  | 4,504 | 0.3908 | 0.4880 |  | 2,356,547 | 0.3306 | 0.4704 |  | 0.1278 |
| Age under 35 |  | 4,504 | 0.0881 | 0.2835 |  | 2,356,547 | 0.1296 | 0.3358 |  | -0.1234 |
| Age 65 and over |  | 4,504 | 0.4545 | 0.4980 |  | 2,356,547 | 0.3807 | 0.4856 |  | 0.1520 |
| Last contacted GP < 6 months ago | | 4,504 | 0.7242 | 0.4469 |  | 2,356,547 | 0.7430 | 0.4370 |  | -0.0430 |
| Last contacted GP > 6 months ago | | 4,504 | 0.2706 | 0.4443 |  | 2,356,547 | 0.2506 | 0.4333 |  | 0.0463 |
| No chronic health condition |  | 4,504 | 0.3521 | 0.4777 |  | 2,356,547 | 0.3449 | 0.4753 |  | 0.0153 |
| Multimorbidity |  | 4,504 | 0.2775 | 0.4478 |  | 2,356,547 | 0.2859 | 0.4519 |  | -0.0186 |
| *Unweighted means. Outcome means for physical functioning, psychological wellbeing, resilience, person centeredness, continuity of care, smoking habit and primary care utilisation normalised to [0,1]. Higher (lower) values indicate better (worse) health status and experience of care, increased (decreased) smoking habit, and higher (lower) primary care utilisation. SMD, standardised mean difference (Cohen's d). Continuity of care has a lower N since many patients indicated they did not have a preferred GP | | | | | | | | | | |
